# Supplementary material for: Genome-wide analysis and characterization of the LRR-RLK gene family provides insights into anthracnose resistance in common bean
Source: Sci Rep. 2023 Aug 18;13:13455. doi: 10.1038/s41598-023-40054-3 (PMC10439169; doi:10.1038/s41598-023-40054-3)
Supplement: Supplementary file 1 — Supplementary Information 1. [file 41598_2023_40054_MOESM1_ESM.pdf]

**Supplementary Figure Legends**

Supplementary Figure S1. Phylogenetic analysis and analysis of functional domains of LRR-RLK proteins from *Phaseolus vulgaris*. In the phylogenetic tree, the different colors represent protein classification into subfamilies obtained by HMMER. The different domains found are also identified by different cores. The figure was created using MEGA-X v.10.2 software (https://www.megasoftware.net) and TBtools v.1.130 software (https://github.com/CJ-Chen/Tbtools/releases).

Supplementary Figure S2. Phylogenetic analysis and analysis of conserved motifs of LRR-RLK proteins from *Phaseolus vulgaris*. In the phylogenetic tree, the different colors represent protein classification into subfamilies obtained by HMMER. The different motifs found are also identified by different cores. The figure was created using MEGA-X v.10.2 software (https://www.megasoftware.net) and MEME Suite v.5.5.3 software (https://meme-suite.org/meme/tools/meme).

Supplementary Figure S3. Phylogenetic analysis and subcellular location heatmap of LRR-RLK proteins from *Phaseolus vulgaris*. In the phylogenetic tree, the different colors represent protein classification into subfamilies obtained by HMMER. The most likely subcellular location is indicated in parentheses next to the protein identification. The figure was created using MEGA-X v.10.2 software (https://www.megasoftware.net) and TBtools v.1.130 software (https://github.com/CJ-Chen/TBtools/releases).

Supplementary Figure S4. Phylogenetic analysis and gene organization analysis, from the exon and intro composition, of LRR-RLK genes from *Phaseolus vulgaris*. In the phylogenetic tree, the different colors represent protein classification into subfamilies obtained by HMMER. The figure was created using MEGA-X v.10.2 software (https://www.megasoftware.net) and GSDS v.2.0 software (http://gsds.cbi.pku.edu.cn/).

Supplementary Figure S5. Phylogenetic analysis and cis-elements analysis of LRR-RLK genes from *Phaseolus vulgaris*. In the phylogenetic tree, the different colors represent protein classification into subfamilies obtained by HMMER. The different cis-elements found are also represented by different colors. The figure was created using MEGA-X v.10.2 software (https://www.megasoftware.net) and TBtools v.1.130 software (https://github.com/CJ-Chen/TBtools/releases).

Supplementary Figure S6. Chromosomal colocalization of *Phaseolus vulgaris* LRR-RLK genes and markers located 500 kb upstream and downstream to anthracnose resistance loci and QTL's. The figure was created using TBtools v.1.130 software (https://github.com/CJ-Chen/TBtools/releases).
