# Supplementary figures and images for: Genome-wide analysis and characterization of the LRR-RLK gene family provides insights into anthracnose resistance in common bean
Source: Sci Rep. 2023 Aug 18;13:13455. doi: 10.1038/s41598-023-40054-3 (PMC10439169; doi:10.1038/s41598-023-40054-3)

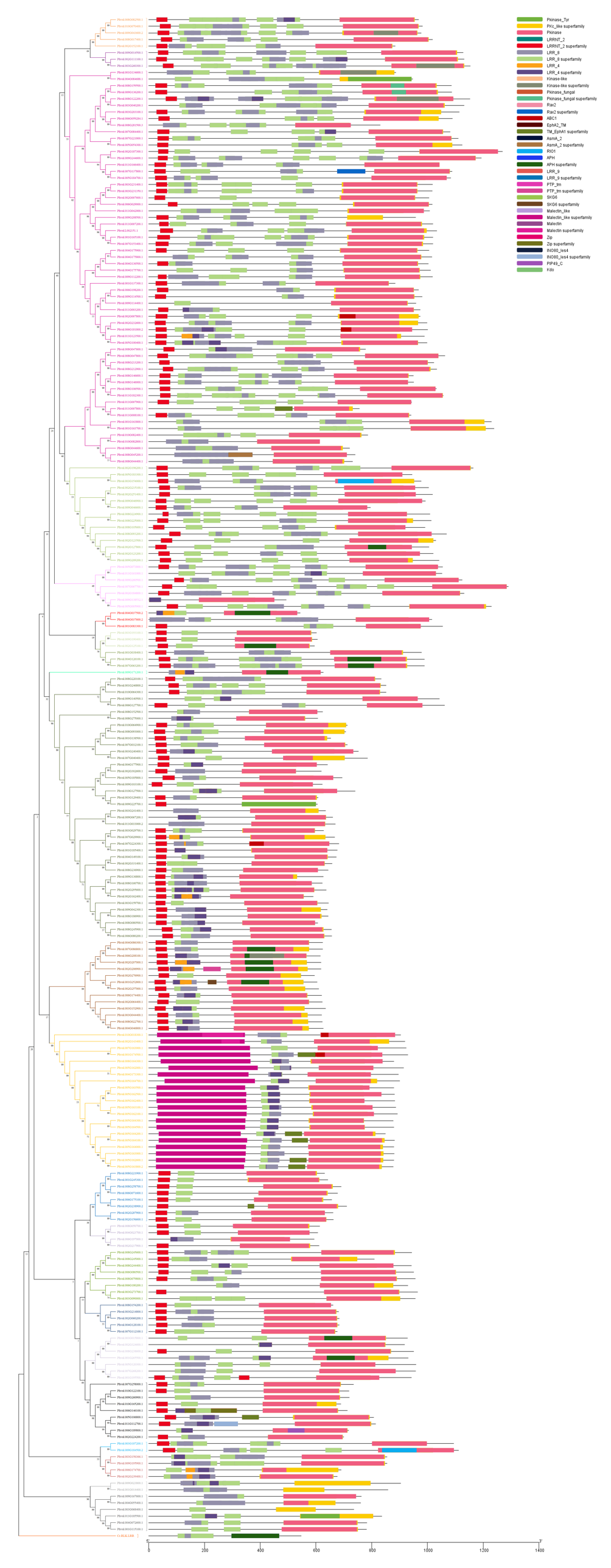

Supplement: Supplementary file 2 — Supplementary Figure S1. [file 41598_2023_40054_MOESM2_ESM.tiff]

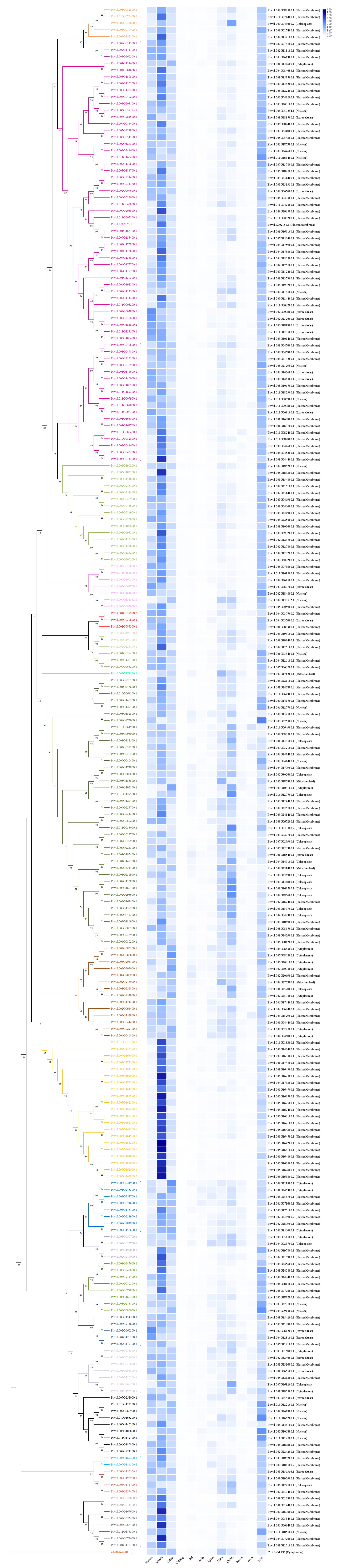

Supplement: Supplementary file 4 — Supplementary Figure S3. [file 41598_2023_40054_MOESM4_ESM.tiff]

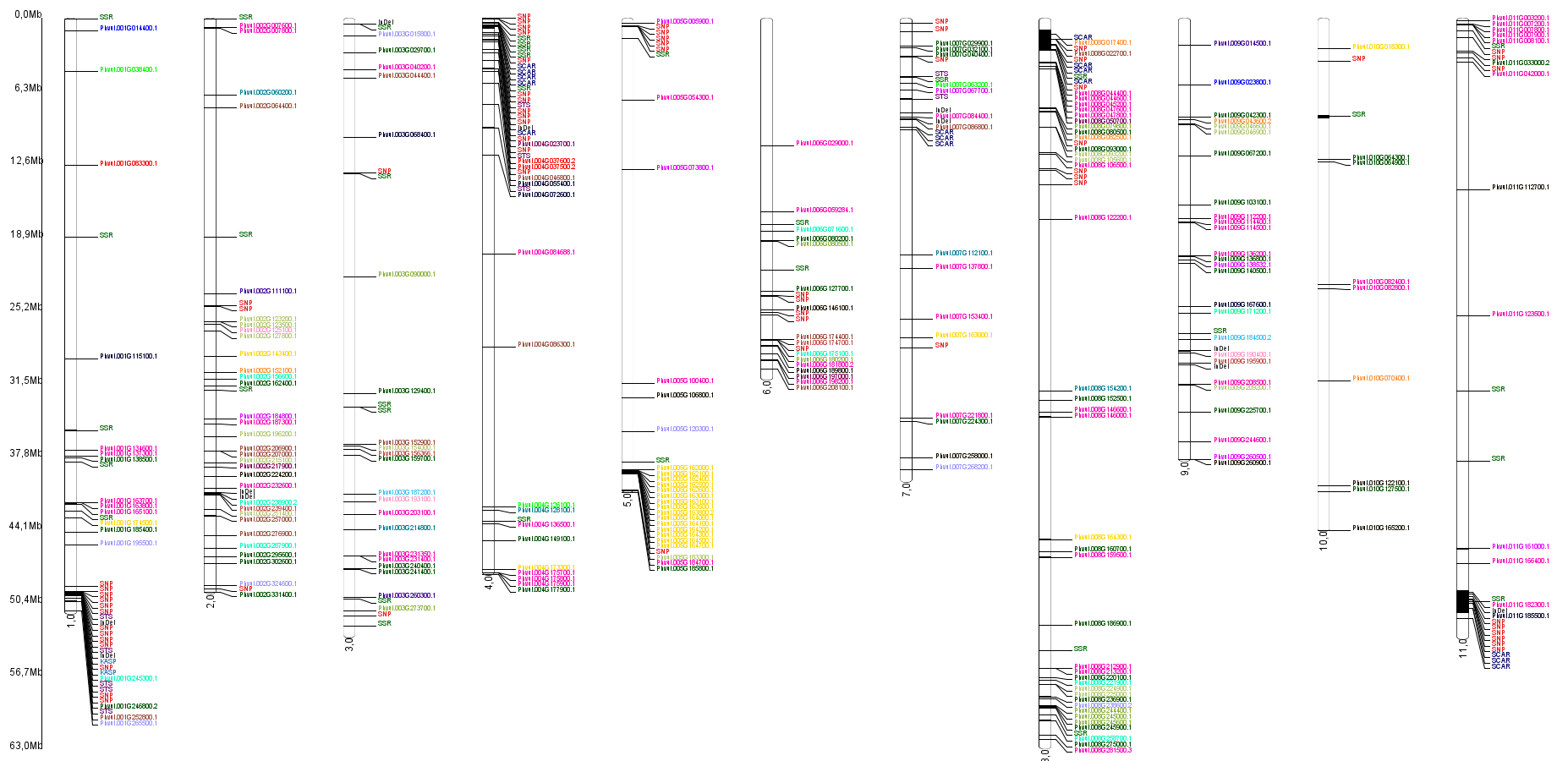

Supplement: Supplementary file 7 — Supplementary Figure S6. [file 41598_2023_40054_MOESM7_ESM.tiff]
